# The Importance of Data Quality Control in Using Fitbit Device Data From the All of Us Research Program

**DOI:** 10.2196/45103

**Published:** 2023-11-03

**Authors:** Lauren Lederer, Amanda Breton, Hayoung Jeong, Hiral Master, Ali R Roghanizad, Jessilyn Dunn

**Affiliations:** 1 Department of Biomedical Engineering, Duke University, Durham, NC, United States; 2 Department of Electrical and Computer Engineering, Duke University, Durham, NC, United States; 3 Vanderbilt Institute for Clinical and Translational Research, Vanderbilt University Medical Center, Nashville, TN, United States

**Keywords:** wearable device, Fitbit, All of Us, data quality, noise, missingness, biometric monitoring

## Abstract

Wearable digital health technologies (DHTs) have become increasingly popular in recent years, enabling more capabilities to assess behaviors and physiology in free-living conditions. The *All of Us* Research Program (AoURP), a National Institutes of Health initiative that collects health-related information from participants in the United States, has expanded its data collection to include DHT data from Fitbit devices. This offers researchers an unprecedented opportunity to examine a large cohort of DHT data alongside biospecimens and electronic health records. However, there are existing challenges and sources of error that need to be considered before using Fitbit device data from the AoURP. In this viewpoint, we examine the reliability of and potential error sources associated with the Fitbit device data available through the AoURP Researcher Workbench and outline actionable strategies to mitigate data missingness and noise. We begin by discussing sources of noise, including (1) inherent measurement inaccuracies, (2) skin tone–related challenges, and (3) movement and motion artifacts, and proceed to discuss potential sources of data missingness in Fitbit device data. We then outline methods to mitigate such missingness and noise in the data. We end by considering how future enhancements to the AoURP’s Fitbit device data collection methods and the inclusion of new Fitbit data types would impact the usability of the data. Although the reliability considerations and suggested literature are tailored toward Fitbit device data in the AoURP, the considerations and recommendations are broadly applicable to data from wearable DHTs in free-living conditions.

## Introduction

Wearable digital health technologies (DHTs) have become increasingly popular in recent years, especially as DHTs offer better user experiences, more capabilities, and greater functionality to assess behaviors and physiology in free-living conditions. The *All of Us* Research Program (AoURP) is an initiative that is seeking to collect health-related information, including DHT data, from a diverse cohort of over 1 million participants in the United States. In the AoURP, DHT data are collected alongside electronic health records, biospecimens, surveys, and standardized physical measurements. The goal is to make these data accessible to both researchers and participants to advance precision diagnosis, prevention, and treatment [1].

In 2019, the AoURP expanded its data collection with the Fitbit Bring-Your-Own-Device (BYOD) project. This expansion has allowed participants to share their historical and ongoing Fitbit account data through the *All of Us* participant portal [2]. The AoURP’s efforts to include Fitbit device data have continued to expand with the WEAR study, which gives eligible participants a no-cost Fitbit Charge 4 or Fitbit Versa 3 device [3]. Data from the BYOD project are available through the AoURP Researcher Workbench, which offers data access and analysis tools for DHT data, electronic health record data, biospecimens, surveys, and physical measurements [4].

As of 2023, over 15,000 *All of Us* participants have shared Fitbit device data [5]. The Researcher Workbench provides access to these data, including Fitbit-defined heart rate by zones that are based on percentages of estimated maximum heart rate, minute-level heart rate, daily activity summaries, minute-level intraday steps, daily sleep summaries, and sleep levels [6].

Digital biomarkers derived from DHTs can potentially be used to improve clinical diagnostics, predict disease status, and support personalized clinical decision-making [7]. With the increasing use of DHTs like Fitbit devices in the AoURP and other research and clinical settings, it is important that those working with these data consider the inherent limitations of Fitbit devices, given their underlying technology. This will enable improved data processing and fit-for-purpose implementations of Fitbit devices in research and clinical settings. Researchers might ask the following: “How reliable is the data from these devices? What are the sources of noise, error, and bias that should be accounted for when using this data? How can these be accounted for?”

In this viewpoint paper, we examine the reliability of Fitbit device data in the context of the AoURP’s BYOD program. We focus on Fitbit devices, given their wide market share [8,9], the ongoing collection of data from Fitbit users in the AoURP (eg, BYOD program) [2,10], and these data’s availability to registered Researcher Workbench users [5]. Data are currently not available from the AoURP regarding the Fitbit device models included in their data set. For this reason, the data cleaning strategies we present are device model agnostic. This paper focuses specifically on data considerations around physical activity (steps and movement intensities) and heart rate measurements generated by Fitbit devices on a daily and per-minute basis. Given that Fitbit device sleep data are derived from the same underlying sensors that determine heart rate and motion intensity metrics, the same fundamental considerations surrounding inherent measurement reliability should be considered when working with Fitbit device sleep metrics.

## Sources of Noise

Measurement error can affect the reliability of Fitbit device data in both laboratory conditions and free-living conditions. In this section, we discuss the most commonly recognized sources of error that may be observed in Fitbit device data collected through the AoURP.

### Inherent Measurement Inaccuracies

Fitbit devices include a 3-axis accelerometer and photoplethysmography (PPG) sensor, with more recent device models including additional sensors, such as an altimeter, a gyroscope, a skin temperature sensor, and multipurpose electrical sensors [11]. It may be helpful for researchers to consider the data supply chain as they work with DHT data [12]; the data that researchers generally have access to are processed, and the firmware that performs such data processing is regularly updated [13]. Aside from the data supply chain, there are also inherent limitations of the accelerometer and PPG sensors themselves that should be accounted for in data analysis.

All Fitbit models with Fitbit LLC’s patented PurePulse technology (eg, Fitbit Charge, Fitbit Charge 2, Fitbit Charge 3, Fitbit Alta, Fitbit Versa, Fitbit Blaze, and Fitbit Ionic) use the same PPG hardware and software for heart rate estimation [14]. PPG sensors, which optically measure light absorption under the skin, may be affected by user motion and activity intensity, skin tone, and the wavelength of light used by the sensor [15,16]. When compared to gold-standard electrocardiography, Fitbit devices tend to underestimate heart rate [14,15,17]. Further, Fitbit device heart rate measurements have higher reliability under stationary conditions [14,18].

Fitbit devices use the 3-axis accelerometer to determine step count and categorize physical activity intensity (sedentary, light, moderate, vigorous, or moderate to vigorous) [19-21]. Comparisons between Fitbit device step counts using direct observation and gold-standard accelerometers, such as the ActiGraph GT3X+, demonstrate mixed reliability, depending on the type and speed of movement and the on-body placement of the Fitbit. During normal walking for example, the torso placement of the Fitbit device has resulted in the greatest accuracy, while ankle and wrist placement have been the most accurate in slow-walking and jogging, respectively [20]. Similar findings from other studies demonstrate that step count and physical activity intensity accuracy are affected by device placement and movement type [22-26].

### Skin Tone

Skin tone may be another inherent source of error for DHTs that rely on optical measurements (eg, PPG or pulse oximetry) [27,28]. Both melanin and skin with tattoos absorb more green light, that is, wavelengths of around 530 nm, which are the LED wavelengths commonly used in PPG sensors [29]. There have been mixed findings in this area; a study by Shcherbina et al [30] on older generations (2014-2016) of consumer smartwatches found that darker skin tones positively correlated with increased heart rate error, whereas our study, in which we used more recent devices (2014-2018), did not find a relationship between heart rate measurement accuracy and skin tone across a subset of consumer smartwatches [18]. Clinical-grade pulse oximeters that rely on red and infrared optical measurement technology may also be affected by skin tone [31].

Fitbit devices may use a combination of both green and red wavelengths to estimate heart rate [15,32]. Although green wavelengths can enable more accurate heart rate measurements during movement when compared to red wavelengths, green wavelengths are more readily absorbed by melanin before reaching the photodetector [29]. Additional research is needed on whether and how the accuracy of optical-based DHT measurements, such as heart rate and saturation of peripheral oxygen (SpO_2_), is affected by skin tone.

The data collected by the AoURP currently do not include data on skin color or the presence of tattoos under Fitbit devices; therefore, it is not possible to directly account for skin tone or wrist tattoos as a potential source of error in AoURP heart rate data. As a result, researchers working with *All of Us* data may need to take extra care when interpreting or translating results that may be influenced by skin tone or the presence of wrist tattoos, particularly when working with heart rate data.

### Movement and Motion Artifacts

Motion artifacts can also be a source of error for heart rate, step count, and physical activity intensity data. Unexpected noise with random amplitudes and frequencies can be seen in raw sensor data and can cause the algorithms of Fitbit devices to falsely detect movement or a heart beat [33]. For example, the reliability of Fitbit Flex’s step count and moderate to vigorous physical activity data was found to be dependent on the activity type (walking, stair stepping, jogging, and incline walking) [24], and step count error was shown to be higher during activity than during rest [20]. Therefore, it is likely that step count reliability varies, particularly during normal household activities, which may be logged by Fitbit devices as exercise movements.

Wearable device heart rate measurements are the most accurate under circumstances of rest, followed by physical activity and then rhythmic activity, such as walking or jogging. Our previous work demonstrated decreased reliability during rhythmic activities, such walking or typing. This was likely due to Fitbit devices mistaking the periodic signal, which was being produced by the repetitive movements, for the cardiovascular cycle. Although walking resulted in heart rate measurements that were higher than the true heart rate, typing resulted in heart rate measurements that were lower than the true heart rate [18]. A study by Benedetto et al [15] assessed Fitbit Charge 2 heart rate accuracy during stationary biking and found that the device underestimated heart rate when compared to electrocardiography.

Some possible reasons for heart rate measurement error during motion include the device’s sampling and interpolation methods, unstable device positioning, and variation in the pressure applied to the skin by the sensor [15,18,34]. Researchers should be aware of the impact of physical activity type (ie, motion intensity and periodicity) on Fitbit device heart rate measurement error.

The body positioning and fit of DHTs can also be sources of motion artifacts. For example, wrist-based Fitbit devices can misclassify nonambulatory arm movements as total body motion, which may result in the overestimation of physical activity and motion intensity [35]. This misclassification of physical activity may be worse if the device is not worn correctly. To address this challenge, the Fitbit device user manuals provide instructions for specific placement on the wrist to enable the acquisition of more reliable data [36].

## Sources of Data Missingness in Fitbit Device Data

It can often be challenging to determine the minimum amount of data necessary to achieve a particular analysis goal when using DHT data. A systematic review by Chan et al [37] pointed to a common definition for a “valid day” of wearable data—at least 10 intermittent hours of data present within 1 day—and a “valid week” of data—at least 3 valid days during the week. It is important for researchers to note that most Fitbit devices need to be charged at least once per week for 1 to 2 hours at the time of writing, and the need to remove the device from the wrist to charge it results in at least some data missingness. More than the minimal necessary data missingness can occur in the event that the wearer forgets to put the device back on their wrist after charging it [35]. Such nonwear is an example of structured missingness, where a contiguous block of missing observations occurs when the device is not being worn. At scale, there may be observable nonwear patterns, such as times when people commonly remove their devices (eg, during sleep) [38].

In addition to nonwear, improper device wear can also result in data missingness. Improper wear, such as insufficient tightening of the wrist strap, can lead to the sensor orientation being askew or a loss of sensor-to-skin contact, which is required for high-fidelity optical measurements, such as PPG-based heart rate measurements [39]. Moreover, observations can be impacted by large motion artifacts, and such observations (eg, high accelerometry values) may be removed by the device firmware. This leads to missing values in the final data set. To explore the extent of such data removal, our team recently compared data missingness in optical heart rate and SpO_2_ observations across multiple wearables [18,40]. We found that, for heart rate measurements, the Fitbit Charge 2 had the highest amount of missing data during both rest (18.7%) and physical activity (10.4%) when compared to other consumer-grade wearables [18]. Data missingness due to improper wear or firmware attempts to account for motion artifacts may be seen in the data set as random missingness, lacking structure and predictability. However, some wearers may be more prone to improper wear or high-intensity activity, which can lead to higher amounts of missing data in individual data sets. Other factors that can affect the presence or absence of Fitbit device data include the frequency of syncing the device with the smartphone app and poor device connectivity [41,42].

There is a taxonomy of mechanisms for missing data, including (1) data missing completely at random (MCAR), where missingness is unrelated to observed characteristics; (2) data missing at random (MAR), where missingness is related to observed characteristics; and (3) data missing not at random (MNAR), where missingness is related to unobserved characteristics. Different methods are required to best account for these three missingness mechanisms during data preprocessing; thus, identifying the type of missingness is an important step in DHT data analysis. In the case of Fitbit device data, observations that are MCAR may be due to nonsystematic device malfunctions, nonsystematic errors in data transfer, or sporadic improper device wear. Observations that are MAR may be the result of a particular device model missing a type of measurement capability (eg, a device that is known to not report heart rate measurements under high-intensity activity). An example of MNAR missingness might be nonwear during a bout of illness or due to a user having a poorly fitting device. In a free-living study, such as the AoURP, all three missingness mechanisms are likely to be present in the Fitbit device data and should be identified and appropriately addressed when possible by, for example, making assumptions about the reasons for the data missingness upon analyzing missingness patterns [43].

## Mitigating Missingness and Noise in DHT Data

### Accounting for Data Missingness

Avoiding data missingness is best done at the data collection stage. Prospective bring-your-own-device studies may increase wear time and improve device fit by incorporating reminders for users to wear the device, adding nonwear alerts for users or the study team, and educating users on fit and charging [44]. It should be noted that AoURP Fitbit device data collection is purely observational and, at this time, does not involve providing any alerts or interventions to improve Fitbit device wear habits.

Some missingness in the data is inevitable. Accounting for data missingness begins by thoroughly identifying the reason for missingness and deciding upon the most appropriate strategy for mitigation. For AoURP researchers using Fitbit device data collected in free-living conditions, it is not always possible to distinguish MAR, MCAR, and MNAR missingness; therefore, they may need to make assumptions to decide how to proceed with mitigation [43]. In the context of Fitbit device data, we can distill a few practical solutions for addressing wear-related structured missingness [38].

The first step is to decide upon a definition for “wear” in the context of the data and the question at hand. Depending on what analysis or question is of interest, the definition of “wear” can drastically change. For example, an analysis involving sleep quality and staging may require the definition of “wear” to include common sleep hours (eg, 8 PM to 8 AM) or a minimum monitored sleep duration time, such as that reported by Fitbit devices, but other analyses may only require a daily wear level, which gives an idea of a participant’s activity and physiological state without requirements regarding wear during specific activities, such as sleep and exercise, as described in the *Sources of Data Missingness in Fitbit Device Data* section [37].

One way to calculate a daily wear level is to leverage minute-level heart rate data, as most consumer devices only collect these data when they are worn on the wrist. This would, for example, help to avoid including step count data that may have been collected when a device is in a purse. By dividing the total count of minute-level heart rate observations collected within a single day by the total number of minutes when such data were possible to collect (1440 min in 1 d), we can derive a reasonable estimate of the proportion of the day when the device was on the wrist.

**Figure 1.**
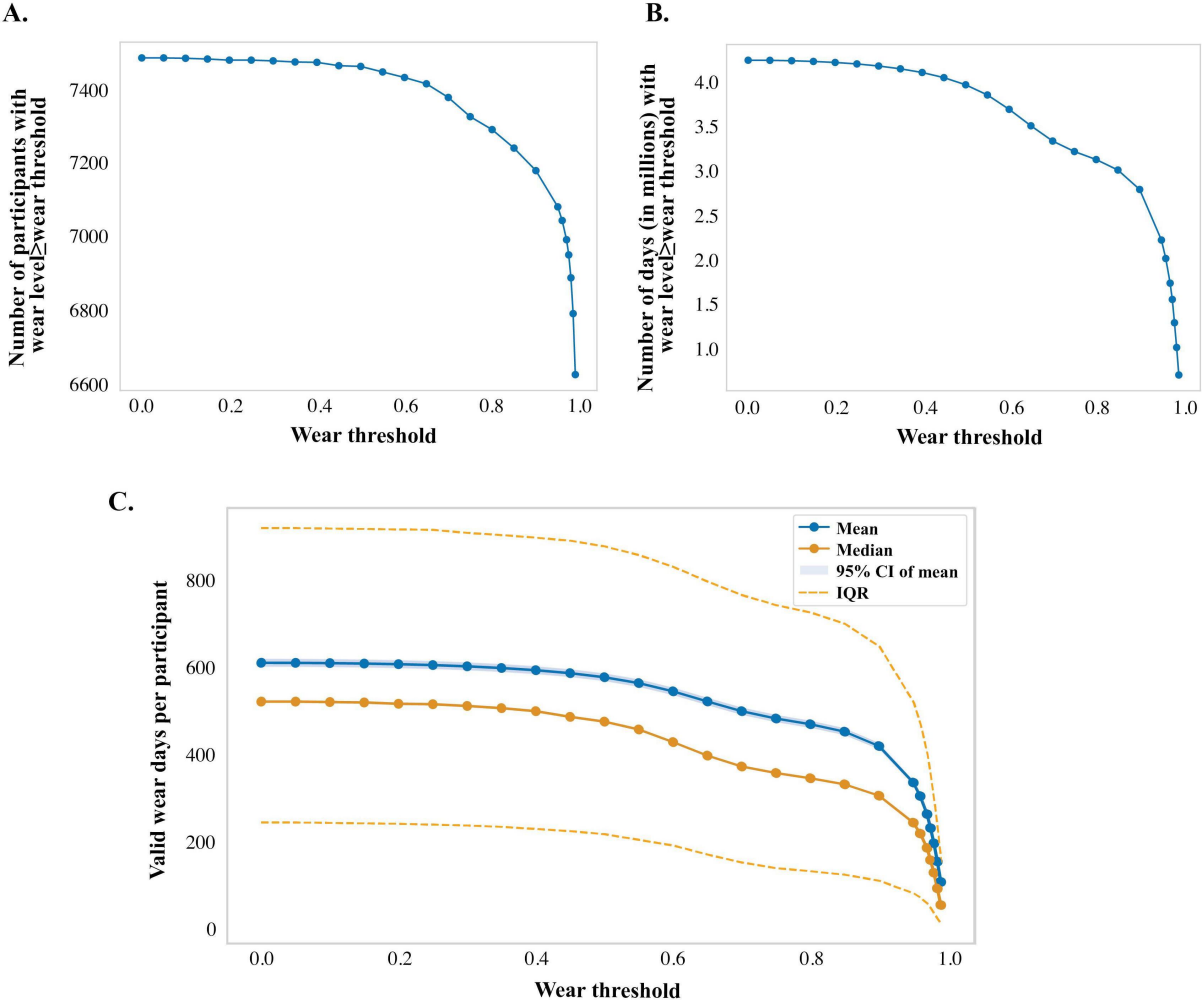
Graphs A and B show the total counts of participants and days, respectively, that meet each wear threshold, with an optimal threshold of <0.4. Graph C shows the mean and median total days (per participant) with a wear level greater than or equal to each wear threshold, demonstrating an optimal threshold of between 0.3 and 0.4. The 95% CI (indicated in blue) was calculated as follows: μ ± σ/√n. The IQR (indicated in orange dashed lines) indicates the first and third quartile values. The controlled tier AoURP version 7 data set (C2022Q4R9) was used to generate Figure 1. AoURP: *All of Us* Research Program.

Once established, the daily wear level threshold can be used for filtering out nonwear data and participants; the optimal threshold for filtering should be selected carefully to avoid unnecessary data loss (Figure 1A, Figure 1B). The optimal threshold is where data loss is minimized and there is adequate statistical power to draw conclusions from the analysis (Figure 1C).

After removing nonwear data (ie, contiguous blocks of missing observations), it is important to identify other sources of data missingness and determine whether mitigation is best done by using imputation or by using the complete case method [43]. Sometimes, the decision can be made based on the extent of the missingness relative to the overall data volume needed for analysis, and at times, it may be deemed that the original analysis cannot be performed as planned due to insufficient data. As an example, a recent study on COVID-19 detection via Fitbit device data calculated the mean over 5-minute intervals of heart rate data; subsequently, any missing data over full 5-minute intervals were imputed by using the median heart rate value from a previously defined 14-day window [45]. Although it is common in wearable data analysis to use the mean and median values of heart rate data for imputation, new imputation methods for biomedical wearable data have also been developed to incorporate machine learning for improved imputation accuracy [46,47]. Although imputation can be beneficial because it preserves data, it should be noted that imputation is not always a good idea, particularly in cases with substantial missingness for which typical values cannot be established.

### Accounting for Noise

Many methods exist to reduce noise in raw signal data (ie, sample-level or high-frequency signal data), particularly when the source of the noise is well characterized. Unfortunately, consumer devices typically do not give access to such high-frequency data, but their firmware and adaptive data collection methods are thought to include steps that account for skin tone–related errors and motion artifacts. Unfortunately, the public has no way of assessing how well these methods perform. Based on this, it is difficult for researchers to directly address errors resulting from skin tone or motion artifact errors. However, some general data cleaning strategies exist that may help to mitigate noise in the data, regardless of the source of the noise (Table 1). In this section, we discuss how to leverage changes from an individual’s baseline, filtering during repeated activities, and *z* score normalization to improve the signal to noise ratio (SNR). We recommend using these techniques in combination with one another to best mitigate noise.

**Table 1.** Examples of noise mitigation methods for wearable data.

	Baseline comparisons	Sampling during periods of similar activity	*z* score normalization
Description	Calculate the median value during a defined baseline period. Calculate the Δ from the baseline for all other data.	Establish specific wear times and use the “Activity Type” metric to filter an individual’s Fitbit device data during similar time periods each day for comparable activity types. Conduct analysis using these segmented data sets.	Subtract the mean from each observation and divide by the SD.
Applicability	Mitigate consistent measurement error (bias).	Mitigate noise that is exacerbated under specific conditions.	Mitigate short periods of noise.
Benefits	Provides a “usual” picture of an individual [48].	Assists in isolating confounding effects that may arise in different activity types and heart rate zones. Standardizes the comparison of data across different individuals.	Allows direct comparison of 2 observations originating from different segments of temporal data [49].
Limitations	Need ample data to establish a baseline [48]. Baselines can change over time.	Recommended for large sets of longitudinal data to make accurate comparisons.	Abstraction of units and range may make it difficult to interpret data.

On an individual level, the comparison of observations to a reliable baseline can be helpful for determining changes in biosignals over time while reducing the influence of both skin tone and motion artifacts. Reliable baselines can be established by first summarizing an individual’s measurements during periods of sleep or inactivity or before a perturbation. The determination of which time period to use to establish a baseline is study dependent. Depending on the timescale of the analysis, it is also useful to consider a sliding window approach, wherein new baselines are established during predefined time periods to account for baseline changes over time. The median value of the biosignal serves as a useful baseline value because it is less susceptible to noise and outliers compared to other statistical summary metrics and provides a way to amplify the SNR during the next steps of the analysis. The establishment of and comparisons to reliable baselines have been performed in multiple studies [50-53]. One limitation of this approach is that substantial monitoring time may be needed to establish a reliable baseline for an individual due to inherent biological and behavioral variability and the effects of external factors that may be difficult to control for (eg, seasonality, circadian rhythms, weekdays vs weekends, etc) [48]. It should also be noted that comparisons to a reliable baseline would not improve the SNR in scenarios where there is a compound effect of the source of noise and the conditions of measurement [54]. For example, skin tone may only increase measurement error for certain heart rate zones (eg, high heart rate) or under circumstances of high motion. In such cases, removing data collected under certain conditions that exacerbate measurement error may be the most appropriate approach.

Another way to handle the challenge of confounding sources of measurement error is to only compare segments of data that are measured under the same conditions (eg, similar movement types and heart rate zones) [15,18,20,24,55]. This technique allows researchers to further isolate confounding sources of measurement error that may be exacerbated during different activities. First, one must define specific wear times of interest, such as wear during specific times of the day, which helps account for circadian variability. Second, when available, researchers should use the “Activity Type” provided by Fitbit devices to segment heart rate data into comparable sections. Researchers can also leverage this activity information to anticipate activities for which heart rate data may be less accurate.

In circumstances where there are short periods of incorrectly reported heart rates or step counts (eg, during high-intensity motion), simple normalization methods, such as *z* score normalization and minimum-maximum normalization, are the most useful [56,57]. Minimum-maximum normalization is useful when extreme outliers are not present in the data, especially when the data have a fixed possible range. *z* score normalization is particularly useful because it centers and scales the Fitbit device data to a mean of 0 and an SD of 1. *z* score normalization helps to reduce the comparatively higher impact of outliers within shorter data segments because it leverages information from longer segments (ie, the mean and SD) for normalization. Once normalized, the data can be compared across wearable data types and participants.

## Future Directions

As the AoURP continues efforts to provide wearable data to researchers and expand the scope of the Fitbit device data made available on the Researcher Workbench, there are several future directions to be considered. Although the ideas presented herein are tailored to Fitbit device data originating from the bring-your-own-device facet of AoURP, Fitbit device data are now actively being collected from other studies, and these data may one day be integrated into the Researcher Workbench [3]. Each additional study may have unique characteristics, including the target population, which may play a role in the overall quality of the data. For example, whether a Fitbit device was provided to participants or whether they were using an existing device they purchased may be a factor in a participant’s comfort level with using the device properly and regularly. Understanding the nuances and potential variations in data quality arising from different study protocols and data sources within the AoURP ecosystem necessitates further research. Investigating how specific study designs, participant demographics, and data collection protocols within the AoURP may influence the overall quality of the collected data will be crucial for researchers seeking to derive meaningful insights and improve the designs of future studies that implement DHTs.

Although Fitbit device model data are not currently available on the Researcher Workbench, it is worth considering how differing device models, software, and firmware may affect the data collected. At the time of writing, the underlying PurePulse PPG technology is the same across all Fitbit LLC heart rate tracking devices [14]. The largest differences in accelerometer-derived data have been observed between Fitbit LLC’s early torso clip-on trackers and the newer wrist-based devices [20]. Additional research is needed to investigate whether there are any substantial differences in accelerometry performance across Fitbit LLC wrist-based models. With regard to data derived from heart rate and accelerometry, such as sleep tracking data, prior to Fitbit LLC’s release of heart rate tracking devices in 2014 [58], Fitbit LLC’s early accelerometry-only devices estimated sleep metrics based on movement alone. Only Fitbit devices with heart rate tracking will include sleep staging, wake heart rate, and sleep-time heart rate [59]. Identifying whether sleep staging metrics are available for a particular individual may be a convenient way to identify the broad type of Fitbit device that was worn. The future incorporation of other contextual information, such as environmental factors, user behaviors, and device models, will enhance the ability to detect and mitigate noise, improve overall data quality, and provide a more comprehensive understanding of an individual’s health.

## Conclusion

The development and validation of Fitbit device–derived digital biomarkers offer the potential for remote and continuous measurement of physiological data. Such digital biomarkers can help inform medical decisions and predict disease states [7]. The wide adoption of DHTs by both consumers and programs like the AoURP make DHTs a great source of data for researchers. Researchers can use various analytical, statistical, and machine learning approaches to further develop DHT data into digital biomarkers [60-63]. Like with any technology, there are inherent limitations and sources of error that stakeholders (eg, researchers using DHT data in their analyses) should be aware of. We encourage the *All of Us* community to use data processing techniques that address noise and missingness to reduce problems downstream in the data analysis. Although we focused on heart rate and motion data in this work, the error mitigation methods described are applicable to other forms of wearable data, including sleep data. For example, changes in total sleep time and sleep stages can be compared against baselines over time. Researchers should consider their study goals and expected outcomes when determining which data cleaning strategies are the most salient to their goals.

## Abbreviations

AoURP: *All of Us* Research Program
BYOD: Bring-Your-Own-Device
DHT: digital health technology
MAR: missing at random
MCAR: missing completely at random
MNAR: missing not at random
PPG: photoplethysmography
SNR: signal to noise ratio
SpO_2_: saturation of peripheral oxygen
